# BCX9250 is a potent small-molecule inhibitor of Activin A Receptor Type 1 (ACVR1/ALK2) for fibrodysplasia ossificans progressiva

**DOI:** 10.1093/jbmrpl/ziag145

**Published:** 2026-08-28

**Authors:** Haitao Wang, Saman Toutounchi, Robert J Pignolo, Xiaogang Cheng, Cynthia Davis Parker, Qingde Liu, Saritha Muppa, Debra Kellogg-Yelder, Kevin J Polach, Pravin L Kotian, Yarlagadda S Babu, Xilin Chen

**Affiliations:** Department of Medicine, Section of Geriatric Medicine & Gerontology, Mayo Clinic, Rochester, MN 55905, United States; Department of Physiology and Biomedical Engineering, Mayo Clinic, Rochester, MN 55905, United States; Department of Medicine, Section of Geriatric Medicine & Gerontology, Mayo Clinic, Rochester, MN 55905, United States; Department of Physiology and Biomedical Engineering, Mayo Clinic, Rochester, MN 55905, United States; Department of Medicine, Section of Geriatric Medicine & Gerontology, Mayo Clinic, Rochester, MN 55905, United States; Department of Physiology and Biomedical Engineering, Mayo Clinic, Rochester, MN 55905, United States; Divisions of Endocrinology and Hospital Internal Medicine, Department of Physiology and Biomedical Engineering, Mayo Clinic, Rochester, MN 55905, United States; Department of Biology, BioCryst Pharmaceuticals Inc., Discovery Center of Excellence, Birmingham, AL 35244, United States; Department of Biology, BioCryst Pharmaceuticals Inc., Discovery Center of Excellence, Birmingham, AL 35244, United States; Department of Biology, BioCryst Pharmaceuticals Inc., Discovery Center of Excellence, Birmingham, AL 35244, United States; Department of Biology, BioCryst Pharmaceuticals Inc., Discovery Center of Excellence, Birmingham, AL 35244, United States; Department of Animal Studies, BioCryst Pharmaceuticals Inc., Discovery Center of Excellence, Birmingham, AL 35244, United States; Department of Biology, BioCryst Pharmaceuticals Inc., Discovery Center of Excellence, Birmingham, AL 35244, United States; Department of Biology, BioCryst Pharmaceuticals Inc., Discovery Center of Excellence, Birmingham, AL 35244, United States; Department of Discovery Chemistry, BioCryst Pharmaceuticals Inc., Discovery Center of Excellence, Birmingham, AL 35244, United States; Department of Biology, BioCryst Pharmaceuticals Inc., Discovery Center of Excellence, Birmingham, AL 35244, United States; Department of Discovery Chemistry, BioCryst Pharmaceuticals Inc., Discovery Center of Excellence, Birmingham, AL 35244, United States; Department of Computational Chemistry and Structural Biology, BioCryst Pharmaceuticals Inc., Discovery Center of Excellence, Birmingham, AL 35244, United States; Department of Biology, BioCryst Pharmaceuticals Inc., Discovery Center of Excellence, Birmingham, AL 35244, United States

**Keywords:** fibrodysplasia ossificans progressiva, FOP, BCX9250, ALK2 inhibition, ACVR1

## Abstract

Fibrodysplasia ossificans progressiva (FOP) is an ultra-rare genetic disease of heterotopic ossification (HO) caused by mutations in the activin receptor-like kinase-2 (ALK2) protein, a BMP type I receptor of the TGF-β superfamily. These mutations in the ALK2 receptor alter receptor function, causing activin A, a protein that normally inhibits ALK2 signaling, to aberrantly trigger suppressor of mothers against decapentaplegic 1/5/8 (SMAD1/5/8) signaling, thereby activating the chondroosteogenic pathway and leading to HO. Here we report the initial characterization of BCX9250, an orally available small-molecule inhibitor of ALK2 developed by BioCryst with the potential to treat FOP. Inhibition of ALK2-mediated signaling by BCX9250 was assessed using biochemical binding assays and in vitro activity assays with the mouse myoblast C2C12 cell line. The impact of BCX9250 on HO was investigated using 2 mouse models and an HO recurrence mouse model. BCX9250 has potent inhibitory activity against wild-type ALK2 protein and mutant ALK2 protein associated with FOP. In the ALK2^R206H^-expressing C2C12 cells, BCX9250 potently inhibited activin A-induced SMAD1 phosphorylation. In the selectivity assays, BCX9250 also potently inhibited several other protein kinases. Oral delivery of BCX9250 significantly reduced HO in 2 mouse models, including the Acvr1^R206H^ knock-in mouse FOP model. BCX9250 also reduced the recurrence of HO after surgical removal of HO, suggesting that ALK2 inhibitors may be beneficial as prophylactic treatments following surgical excision of HO. These results suggest that BCX9250 may be useful for the treatment of FOP.

## Graphical Abstract

**Graphical Abstract:**
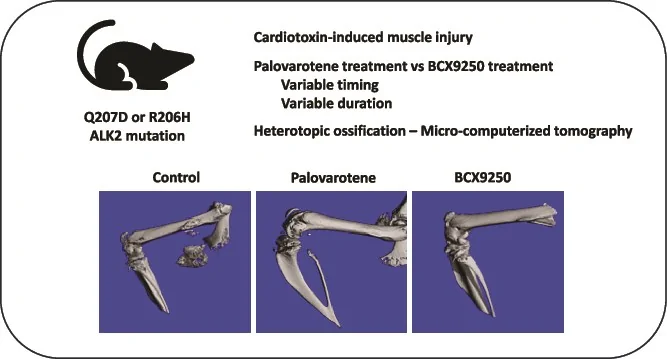

## Introduction

Fibrodysplasia ossificans progressiva (FOP) is an ultra-rare genetic disorder characterized by heterotopic ossification (HO) in skeletal muscle, tendons, ligaments, and fascia.^1–3^ The onset of symptoms usually occurs during early childhood, with episodes of painful swelling, inflammation, and stiffness (described as flare-ups) that can occur spontaneously or be triggered by trauma, infection, or muscle overuse.^2^^,^^4^^,^^5^ Heterotopic ossification leads to skeletal deformities, joint immobility, decreased quality of life, and decreased life expectancy.^6–9^ Surgical excision of the heterotopic bone is contraindicated in FOP patients due to the trauma of the surgery, which can trigger inflammation and recurring HO that undermine the intervention.^10^^,^^11^ Current treatments do not prevent disease progression, and there is currently no cure; however, newer therapeutics may provide beneficial alterations to the natural course of the condition.^4^^,^^12–14^

Fibrodysplasia ossificans progressiva is caused by mutations in the activin receptor-like kinase-2 (ALK2) receptor that alter its function, leading to aberrant activation of suppressor of mothers against decapentaplegic 1/5/8 (SMAD1/5/8) signaling by activin A, a protein that normally inhibits ALK2 signaling, thereby activating the chondroosteogenic pathway and resulting in HO. Activin A binds and uniquely activates FOP-mutant ALK2 protein (encoded by the Activin receptor A type I [*ACVR1*] gene^2^^,^^15^^,^^16^) and drives HO. The 14 known FOP-associated ALK2 mutations lie within the intracellular portion of the receptor, in either the glycine-serine-rich (GS) domain or the protein kinase (PK) domain.^6^^,^^7^^,^^17^ Greater than 95% of all FOP cases result from 1 specific mutation, a single nucleotide substitution at position 617 (617G > A) that replaces arginine 206 with a histidine (R206H) within the GS domain.^16^^,^^17^ In receptor complexes containing the ALK2 FOP mutant proteins, Binding of activin A to its receptors activates the SMAD1/5/8 signaling pathway,^18–20^ stimulating chondrogenesis.^21–24^ Inhibition of activin A in mouse models of FOP halts the HO process,^22^ and Garetosmab (REGN247, an activin A-neutralizing antibody) reduces flare-ups and ALK2-mediated activation of HO in FOP patients.^25^^,^^26^

Inhibitors targeting other SMAD1/5/8 signaling components (the mutant ALK2 receptors, in particular) can also prevent HO in mouse models of FOP, providing a pathway for developing potential therapeutics.^4^^,^^22^^,^^27–31^ Palovarotene is a selective agonist of retinoic acid receptor gamma that has been shown to effectively reduce HO and maintain limb mobility and growth in mice with the human R206H FOP mutation.^4^ Palovarotene reduces whole body HO in FOP patients and is the first FDA-approved drug for FOP.^32^^,^^33^ BioCryst has discovered and developed BCX9250, a novel small molecule that can inhibit human activin A and ALK2-SMAD1/5/8-mediated signaling. This study assessed the potency of BCX9250 as an ALK2 inhibitor and evaluated its effectiveness in reducing both initial and relapsing HO (following surgery) in a mouse model of FOP.

## Materials and methods

### BCX9250

The identification, synthesis, purification, and chemical structure of BCX9250, together with additional characterization of its pharmacokinetic properties, in vivo target engagement, and safety profile, will be reported separately.

### Cell culture and animal models

The C2C12 mouse myoblast cell line was grown in DMEM/Nutrient Mixture F-12 (DMEM/F12) medium with 10% FBS supplemented with 1% penicillin-streptomycin.

All studies involving animals adhered to the Animal Welfare Act (9 CFR Parts 1-3). Furthermore, when the studies were conducted in-house, we followed the Guide for the Care and Use of Laboratory Animals, Institute of Laboratory Animal Resources, National Academy Press, Washington, DC. We also adhered to all internal regulations when the studies were conducted at an outsourced facility. The study protocols underwent rigorous review and were approved by the Executive Director of Research Biology at BioCryst and the Institutional Animal Care and Use Committee at the Mayo Clinic, Rochester, MN, ensuring that animal welfare was upheld. Mice were housed in the animal care facility (gang housing), husbandry conditions were pathogen-free, and the standard mouse diet was Lab Diet 5053 irradiated pellets (catalog number 3005740-220).

The Q207D mouse is a transgenic mouse model containing an *Acvr1* allele flanked by loxP sites.^34^ To induce expression of *Acvr1*, 20 μL of a solution of adenovirus-Cre (5 × 10^10^ genome copies per mouse diluted in 0.9% NaCl) was injected i.m. into C57BL/6 mice (female/male; 3 wk old; 10-15 g). To induce an injury/inflammatory response, 100 μL of cardiotoxin (i.m.; 10 μM, in 0.9% NaCl; Sigma-Aldrich, St. Louis, MO, USA) was injected into the left hind limb musculature of C57BL/6 mice (female/male; 3 wk old; 10-15 g). The formation of heterotopic bone was monitored by micro-CT (μCT) 14 d after the adenovirus-Cre/cardiotoxin injection.

The FOP *Acvr1*  ^huR206H-Ki/+^ knock in mice were gifted by Dan Perrien and distributed by the International FOP Association (IFOPA) .^35^The *Acvr1*  ^huR206H-Ki/+^ genotype was confirmed by PCR. 

### μCT scans and imaging analysis

We used μCT to confirm the presence of HO, measure the volume of heterotopic bone, and obtain medial sagittal plane images of each limb. The scans were performed with an Inveon scanner (Siemens, Germany) using the following settings: 80 kVp, 80 mA, and 1.1 s of exposure. To distinguish bone from non-bone tissue, we used an upper threshold of 1000 Hounsfield units and a lower threshold of 180 Hounsfield units. We quantified the amount of heterotopic bone using the V6.6 micro-CT evaluation software, manually selecting each ROI.

### Inhibition of the binding activity of human activin receptor-like kinase-2

To determine the IC_50_ of BCX9250 inhibition of the binding activity of purified ALK2^WT^ and ALK2^R206H^, we performed a LanthaScreen Eu Kinase Binding Assay (ThermoFisher Scientific, Madison, WI). An anti-glutathione-S-transferase (GST) Eu-labeled antibody binds to GST-tagged ALK2^WT^ or ALK2^R206H^ proteins, followed by an Alexa Fluor conjugated probe that binds to the ATP binding sites. Proximity of the 2 fluorescent molecules in the complex results in a FRET signal that is decreased upon binding of the BCX9250 kinase inhibitor, which displaces the fluorescent probe. BCX9250 was mixed with the ALK2 kinase, Eu anti-GST antibody, tracer 236, and kinase buffer in a white 384-well plate (total volume of 16 μL per well) for 30 s, followed by a 60-min incubation at room temperature. Fluorescence was measured using a plate reader, then emission ratios (ERs) and values for percentage of displacement were calculated using equations:


$$ \mathrm{ER}=\frac{\mathrm{Alexa}\ \mathrm{Fluor}\ \left(\mathrm{AF}\right)\ 647\ \mathrm{emission}\ \mathrm{at}\ 665\ \mathrm{nm}}{\mathrm{Europium}\ \mathrm{emission}\ \mathrm{at}\ 615\ \mathrm{nm}} $$

The % displacement (of tracer) by BCX9250 was determined with the following equation:


\begin{align*}&\kern-3pt \left\{\frac{\mathrm{ER}\ \left(0\%\ \mathrm{displacement}\ \mathrm{control}\right)\hbox{--} \mathrm{ER}\ \left(\mathrm{sample}\right)}{\!\!\mathrm{ER}\ \left(0\%\, \mathrm{displacement}\ \mathrm{control}\right)\hbox{--} \mathrm{ER}\ \left(100\%\, \mathrm{displacement}\ \mathrm{control}\right)\!\!}\right\}\\&\qquad\ast 100 \end{align*}

SelectScreen Kinase Profiling Services used XL*fit* from IDBS (Boston, MA). The dose-response curve was curve fitted to Model Number 205 to determine the IC_50_ value.

### Inhibition of activin A and ALK2 ^R206H^-mediated phosphorylation of SMAD1 in C2C12 cells by BCX9250

C2C12 cells were plated at a density of 3 × 10^4^ cells/well in a 96-well plate and infected with adenovirus expressing FOP mutant Ad.ALK2^R206H^ using a multiplicity of infection of 5000 for 24 h. After infection, the cells were pretreated with BCX9250 in a half-log dilution series with final concentrations ranging from 1 to 1000 nM, or with 1% DMSO as a control, for 1 h before being treated with activin A (50 ng/mL) for 30 min.

To determine phosphorylated SMAD1 (pSMAD1) levels, C2C12 cell extracts were prepared, and the AlphaLISA SureFire Ultra p-SMAD1 (Ser463/465) Assay Kit was used according to the manufacturer’s instructions. AlphaLISA activity was recorded with a Synergy Neo microplate reader (BioTek, Winooski, VT).

### Determination of the IC_50_s of BCX9250 against BMP-regulated kinases ALK1, ALK2, ALK3, and ALK6

The half maximal inhibitory concentration (IC_50_) values of BCX9250 against ALK1, ALK2, ALK3, and ALK6 were determined in C2C12 cells using cell-based, kinase-mediated transcriptional activity assays. Initially, C2C12 cells were seeded at 3 × 10^4^ cells/well in 96-well plates with DMEM/F12 medium containing 0.1% FBS. Then, the cells were transiently transfected with constitutively active (ca) ALK1, ALK2^R206H^, caALK3, or caALK6 expression vectors and a BMP-responsive element luciferase (BRE)-Luc construct. The BRE-Luc construct includes regions of the mouse inhibitor of DNA binding 1 (Id1) promoter fused to a luciferase reporter gene, which is crucial for BMP-induced Id1 transcription. The transfections were performed using Lipofectamine 3000 (ThermoFisher) according to the manufacturer’s instructions with 100 ng of DNA per well for each vector. Subsequently, the C2C12 cells were treated with BCX9250 or 1% DMSO for 1 h after transfection. The BCX9250 was added in a half-log dilution series with final concentrations ranging from 1 nM to 1000 nM. The C2C12 cells were then incubated at 37 °C for 24 h, and the luciferase activity was measured using the Bright-Glo reagent (Promega) according to the manufacturer’s instructions. The luminescence activity was recorded using a BioTek Synergy Neo plate reader (BioTek, Winooski, VT). The BRE-driven luciferase activity was used to indicate ALK1, ALK2, ALK3, and ALK6-mediated transcriptional activity.

### Determination of the IC_50_s of BCX9250 against TGF-β-mediated transcriptional activity assays

The Cignal SMAD Luciferase Reporter Assay (Qiagen) reports on the combined cellular signaling activity of ALK4, ALK5, and ALK7, where the reporter plasmid expresses luciferase in response to TGF-β. C2C12 cells were transfected with 100 ng of DNA per well using Lipofectamine 3000 (ThermoFisher) according to the manufacturer’s instructions. One hour after transfection, cells were treated with BCX9250 (1-1000 nM) or 1% DMSO. Following this, cells were treated with 2 ng/mL of TGF-β and incubated at 37 °C for 24 h. TGF-β-driven luciferase activity was measured as described earlier and used as an indicator of TGF-β pathway-mediated transcriptional activity.

### Biochemical assays of BCX9250 kinase inhibitory activity

To evaluate BCX9250’s inhibitory activity in a panel of 48 protein kinases, ThermoFisher SelectScreen Kinase Profiling Service (Madison, WI) was utilized. The kinases were chosen from the kinome tree and were evaluated at 1.0 μM BCX9250. Kinases that were inhibited by 80% or greater were further assessed as a function of inhibitor concentration so that IC_50_s could be determined for each.

### Hepcidin and pSMAD levels in Q207D and R206H mouse models with BCX9250 treatment

The *Acvr1^Q207D^* mice and *Acvr1^R206H^* mice received an injection of cardiotoxin in the left hind limb and received either water (vehicle control, per os [by mouth, PO] administration, 5 mice), or multiple treatments with BCX9250 over 14 d (PO, 120 mg/kg, bis in die [2 times a day, BID], 10 mice). Serum and plasma samples were prepared from BCX9250-treated and control animals for hepcidin analysis using a Hepcidin Murine-Compete ELISA (Intrinsic Lifesciences). Serum or plasma samples were prepared and the ELISA was performed according to the manufacturer’s instructions (1:20 dilutions of the serum and plasma samples). Activity was recorded with a Synergy Neo microplate reader (BioTek, Winooski, VT).

Tissues were fixed in 4% paraformaldehyde and embedded in paraffin. Sections (7 μm thick) were deparaffinized in a graded series of ethanol and stained with hematoxylin and eosin (H&E) and immunohistochemically with p-SMAD1/5 antibody [1:500 dilution for overnight at 4 °C (cell signaling, 9516)]. Signal was detected per instructions outlined in the Vectastain Elite ABC kit (Vector Laboratories, PK-6101), and using the DAB Peroxidase Substrate (Vector Laboratories, PK-4100).

### Evaluating the efficacy of BCX9250 in Q207D and R206H mouse models

The *Acvr1^Q207D^* mice received an injection of cardiotoxin in the left hind limb and were divided into 4 groups (5 mice/group): Group 1 (control group) received 100 μL of water PO for 15 d; Group 2 received palovarotene PO (2 mg/kg) once a day (quaque die [once a day, QD]) for 15 d; Group 3 received BCX9250 PO (120 mg/kg) BID for 15 d; Group 4 received BCX9250 PO (120 mg/kg, ter in die [3 times a day, TID]) for 15 d.

The *Acvr1^R206H^* mice were divided into 3 groups (5 mice/group): Group 1 (control) received 100 μL of water PO for 15 d; Group 2 received palovarotene PO (2 mg/kg QD) for 15 d; Group 3 received BCX9250 PO (120 mg/kg BID) for 15 d.

Assessments were made for weight loss, alopecia, gross visual problems, and altered activity of the mice following dosing. After 15 d, mice were euthanized, tissues were harvested, and samples were analyzed by μCT analysis.

### Evaluating the efficacy of BCX9250 in the R206H mouse model following early treatments and delayed treatments

On day 0, R206H mice received an injection of cardiotoxin in the left hind limb as described above and were divided into 4 groups (5 mice/group): Control group (vehicle treatment) received H_2_O PO (BID) from day 1 through day 21; Group 1 (early treatment) received BCX9250 PO (120 mg/kg BID) from day −1 through day 5; Group 2 (delayed treatment) received BCX9250 PO (120 mg/kg BID) from day 3 through day 21; Group 3 (delayed treatment) received BCX9250 PO (120 mg/kg BID) from day 7 through day 21. Tissues were harvested for analysis by μCT on day 21.

### Testing BCX9250-mediated inhibition of relapsing HO in the R206H mouse model

The relapsing HO model was established using the *Acvr1^R206H^* mouse model of FOP. On day 0, R206H mice received an injection of cardiotoxin in the left hind limb as described above. On day 21, we surgically removed the formed HO. After restitching the open cut, we used X-ray imaging to confirm the HO removal, then monitored the mice for 21 d following the postsurgical live animal care procedure at Mayo Clinic.

The efficacy of BCX9250-mediated inhibition of relapsing HO was assessed following surgery to remove the initially formed HO. Mice were divided into 2 groups (5 mice/group): Group 1 (control group) received 100 μL of water PO for 21 d; Group 2 received BCX9250 PO (120 mg/kg BID) from day 21 through day 42. The mice were monitored for 21 d following the postsurgical live animal care procedure at Mayo Clinic. At the end of 21 d, we euthanized the mice and harvested the leg for CT imaging analysis. Relapsing HO was indicated by newly formed HO; the volume of newly formed HO was quantified using μCT analysis.

### Statistical analysis

For the in vitro studies, IC_50_s were calculated using either SigmaPlot (version 11; SPSS, Chicago, IL) or GraphPad Prism software (version 8; Boston, MA) by fitting data to a 4-parameter, nonlinear regression equation. Data are represented as means ± SE.

For the in vivo studies, 1-way ANOVA was used for multiple groups with Tukey’s multiple comparisons, or a T-test was used for 2-group comparisons. Both were set with a significant level of 0.05. Data are shown as a scatter plot with average. GraphPad Prism, version 8, was used for all statistical analyses.

### Data availability

All relevant raw data have been incorporated into the article.

## Results

### BCX9250 binding to purified wild-type ALK2 and ALK2^R206H^

**Figure 1:**
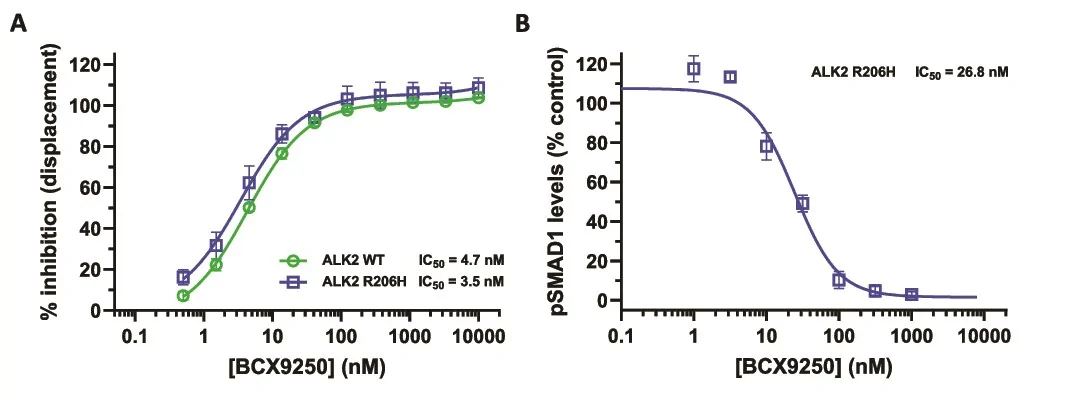
BCX9250 binding to purified activin receptor-like kinase-2 (ALK2) and ALK2^R206H^, and BCX9250 inhibition of activin A-induced suppressor of mothers against decapentaplegic 1 (SMAD1) phosphorylation. (A) Binding of BCX9250 to ALK2^WT^ (*N* = 4) and the fibrodysplasia ossificans progressiva-mutant ALK2^R206H^ (*N* = 3) as measured by a competitive fluorescent ligand binding assay; (B) C2C12 cells were infected with Ad.ALK2^R206H^ and treated with activin A. BCX9250 inhibited activin A-induced phosphorylation of endogenous SMAD1 in ALK2^R206H^-expressing cells (*N* = 4).

To be effective, an inhibitor must bind its target molecule with high affinity. BCX9250 displaced the ALK2 probe from both wild-type ALK2 and ALK2^R206H^ in a concentration-dependent manner, with similar IC_50_s observed for each (4.7 nM for ALK2^WT^ and 3.5 nM for ALK2^R206H^, Figure 1A).

### BCX9250 inhibition of activin A-mediated the SMAD1/5/8 signaling

To evaluate BCX9250-mediated inhibition of activin A-mediated SMAD1/5/8 signaling, C2C12 cells were infected with an adenovirus expressing ALK2^R206H^ (Ad.ALK2^R206H^) to enable C2C12 to respond to activin A. Treatment of ALK2^R206H^ expressing C2C12 cells with activin A resulted in activation (phosphorylation) of the downstream signaling molecule, SMAD1, which was inhibited by BCX9250 with an IC_50_ of 26.8 nM (Figure 1B).

### Selectivity of BCX9250 inhibition of ALK1-, ALK2-, ALK3-, ALK6-, and TGF-β-mediated signaling in C2C12 cells

The BMP-responsive luciferase assay was used to assess the selectivity of BCX9250-mediated inhibition of ALK signaling through ALK1, ALK2, ALK3, and ALK6. C2C12 cells were co-transfected with plasmids expressing ca mutants of each ALK protein and a BRE-Luc reporter plasmid. BCX9250-mediated inhibition was less potent with these kinases when compared to its inhibition of ALK2^R206H^ (IC_50_ = 35.8 nM in this assay), with IC_50_s reflecting a 4.2-fold lower potency for caALK1 and caALK3, and a 14.1-fold lower potency for caALK6. The Cignal SMAD Reporter Assay was used to evaluate BCX9250-mediated inhibition of TGF-β signaling through a TGF-β-driven luciferase reporter plasmid. In this experimental system, exogenous TGF-β activates endogenous receptors (ALK4, ALK5, and ALK7) in place of overexpressed ca receptors. C2C12 cells transfected with the reporter plasmid and treated with increasing concentrations of BCX9250 showed less potent inhibition of luciferase expression, consistent with less potent inhibition of the ALK4-, ALK5-, and ALK7-mediated signaling (IC_50_^SMAD Luc^ = 652 nM, Table 1).

**Table 1:** BCX9250 inhibition of the ALK1, ALK2, ALK3, ALK6, and TGF-β signaling pathways.

**Enzyme/pathway**	**IC** _**50**_ ^**BRE-Luc**^ **± SE (nM)**	**Fold reduction in potency (relative to IC** _**50**_ ^**BRE-Luc**^ **of ALK2** ^**R206H**^ **)**
**ALK2** ^**R206H**^	35.8 ± 5.1	–
**caALK1** ^**Q201D**^	151.3 ± 51.1	4.2
**caALK3** ^**Q233D**^	151.0 ± 73.7	4.2
**caALK6** ^**T194D**^	503.8 ± 79.7	14.1
**TGF-β** ^**a**^	652.0 ± 80.1	18.2

Abbreviations: ALK, activin receptor-like kinase (1, 2, 3, or 6); BRE-Luc, BMP-responsive element luciferase; IC_50_, 50% of the maximal inhibitory concentration.

a Inhibition of TGF-β ligand-induced luciferase activity results expressed in mean ± SE. Means represent 4 independent studies performed in duplicate.

### Selectivity of BCX9250-mediated inhibition of human kinases

BCX9250-mediated inhibition of other human protein kinases was assessed using the ThermoFisher SelectScreen Kinase Profiling Service (Madison, WI). Of the 48 kinases evaluated (Table 2), 11 exhibited more than 80% inhibition at a concentration of 1.0 μM BCX9250. These 11 kinases (along with ALK2, ALK4, and ALK5) underwent additional testing to determine the IC_50_ values (ranging from 1.49 to 1080 nM), which were used to calculate the fold reduction in potency relative to ALK2. Two of these kinases (LCK and SRC) were inhibited by BCX9250 more potently than ALK2 (IC_50_ values of 2.53 nM and 1.49 nM, respectively), while a third kinase (RIPK2) showed a similar level of inhibition (IC_50_ value of 4.49 nM).

**Table 2:** BCX9250 inhibition of human protein kinases.

**Kinase name**	**% Inhibition (mean)** **(1 μM BCX9250)**	**IC** _**50**_ **(nM)**	**Fold reduction in potency (relative to IC** _**50**_ **of ALK2)**
**ALK2**	–	4.7	1.0
**ALK4**	–	1080	229.8
**ALK5**	–	48.2	10.3
**ACVR2A**	111	19.9	4.2
**ABL1**	106	12.1	2.6
**BLK**	103	10.5	2.2
**LCK**	103	2.53	0.5
**RIPK2**	100	4.49	1.0
**TGFBR2**	99	97.3	20.7
**EPHB1**	99	35.1	7.5
**SRC**	98	1.49	0.3
**BTK**	96	25.4	5.4
**CSK**	88	135	28.7
**MINK1**	87	401	85.3

Biochemistry determination of BCX9250 inhibition of selected protein kinases was performed by ThermoFisher SelectScreen Kinase Profiling Service. The full screen included the following enzymes: ALK2, ALK4, ALK5, ACVR2A, ABL1, BLK, LCK, RIPK2, TGFBR2, EPHB1, SRC, BTK, CSK, MINK1, AURKA (Aurora A), TAOK2 (TAO1), JAK2, MAPK11 (p38 beta), MAP3K2 (MEKK2), MST4, PAK1, STK33. MARK2, CLK2,B, MPR2, NUAK1 (ARK5), PDK1, TBK1, DYRK2, GSK3B (GSK3 beta), CHEK2 (CHK2), ALK, CSNK1A1 (CK1 alpha 1), DAPK1, MAPK3 (ERK1), FGFR1, NEK2, IGF1R, PRKCA (PKC alpha), MELK, AMPK A1/B1/G1, SRPK1, HIPK1 (Myak), CAMKK2 (CaMKK beta), SYK, BRSK2, CSNK2A1 (CK2 alpha 1), PHKG1, ZAP70, CAMK1 (CaMK1), and MAPK8 (JNK1).

### BCX9250 increased hepcidin levels in the Acvr1 ^Q207E^ mouse model

**Figure 2:**
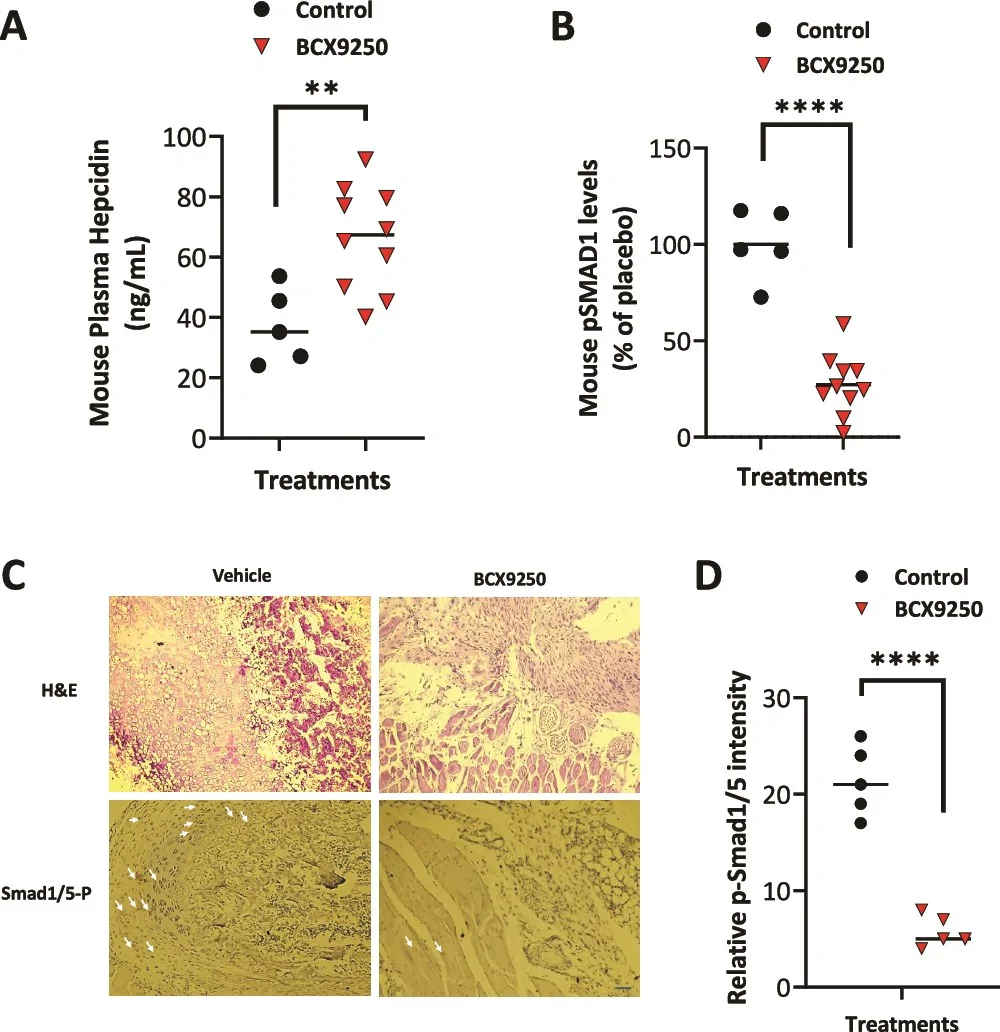
Hepcidin levels and suppressor of mothers against decapentaplegic 1 (SMAD1) phosphorylation following BCX9250 treatment in *Acvr1^Q207D^* and *Acvr1^R206H^* mice. (A) Hepcidin levels in mouse plasma from the *Acvr1^Q207D^* mice following 14 d of BCX9250 treatment (per os [by mouth, PO], 120 mg/kg, bis in die [2 times a day, BID]; *N* = 5 for control mice, *N* = 10 for BCX9250-treated mice); (B) phosphorylated SMAD1 (pSMAD1) levels in mouse livers from the *Acvr1^Q207D^* mice following 14 d of BCX9250 treatment (PO, 120 mg/kg, BID; *N* = 5 for control mice, *N* = 10 for BCX9250-treated mice); (C) sections from *Acvr1^R206H^* mouse muscles (7 μm thick) stained with H&E (top panels) and immunohistochemistry images showing p-SMAD1/5 (brown staining, arrows, bottom panels) in CTX-treated mice (left panels) and BCX9250-treated mice (right panels) at different time points (*N* = 5 for control mice, *N* = 10 for BCX9250-treated mice); scale bar = 25 μm; (D) relative pSMAD1/5 intensity levels (*N* = 5 for control mice, *N* = 5 for BCX9250-treated mice). All statistical analyses were performed by unpaired *T* test; ^**^p < .01; ^****^p < .0001.

ALK2 inhibition can cause reductions in hepcidin expression and disruptions in iron homeostasis, which can lead to iron overload.^36^^,^^37^ A hepcidin ELISA kit was used to evaluate the potential impact of BCX9250 treatment on hepcidin levels, monitoring plasma levels in the *Acvr1^Q207D^* model mice following dosing with BCX9250 (PO administration at 120 mg/kg). Following 14 d of BCX9250 treatment (PO administration at 120 mg/kg, BID [2 times a day]), hepcidin plasma levels were higher in the BCX9250-treated mice (66 ng/mL) than in the placebo-treated mice (37 ng/mL, *p* < .001; Figure 2A).

### BCX9250-mediated inhibition of SMAD1 phosphorylation

A pSMAD-specific ELISA kit was used to monitor SMAD1 phosphorylation levels in liver tissues following oral dosing with BCX9250 (PO administration of 120 mg/kg, BID). pSMAD1 levels decreased in the *Acvr1^Q207D^* model mice following PO administration of BCX9250 at 120 mg/kg, dropping to 27% of the mean level observed in control mice (Figure 2B). pSMAD1/5 levels were also assessed in muscle tissues from Acvr^R206H^ model mice by immunohistochemistry using a pSMAD1/5 antibody (Figure 2C and D). The relative pSMAD1/5 intensity levels were reduced in the BCX9250-treated animals compared to control animals.

### Treatment with BCX9250 reduces HO in mouse models

Two mouse models were used to evaluate BCX9250-mediated inhibition of HO. The *Acvr1^Q207D^* model^34^ is a ca mutation that promotes downstream SMAD1/5/8 signaling independent of ligand binding. While this specific mutation provides an excellent reporter through strong activation of this signaling pathway, it does not occur in FOP patients.^38^ The *Acvr1^R206H^* model, in contrast, utilizes the most frequently occurring mutation in FOP patients.^23^ Exploratory studies investigating the efficacy of BCX9250 in 2 mouse models of FOP suggested decreases in HO following multiple doses at lower drug levels, though these decreases were not statistically significant (data not shown). These results guided the 120 mg/kg dose, which was chosen as the highest safe dose that would provide efficacy in the mouse model systems. Future dose-ranging studies are needed to determine the lowest efficacious dose.

**Figure 3:**
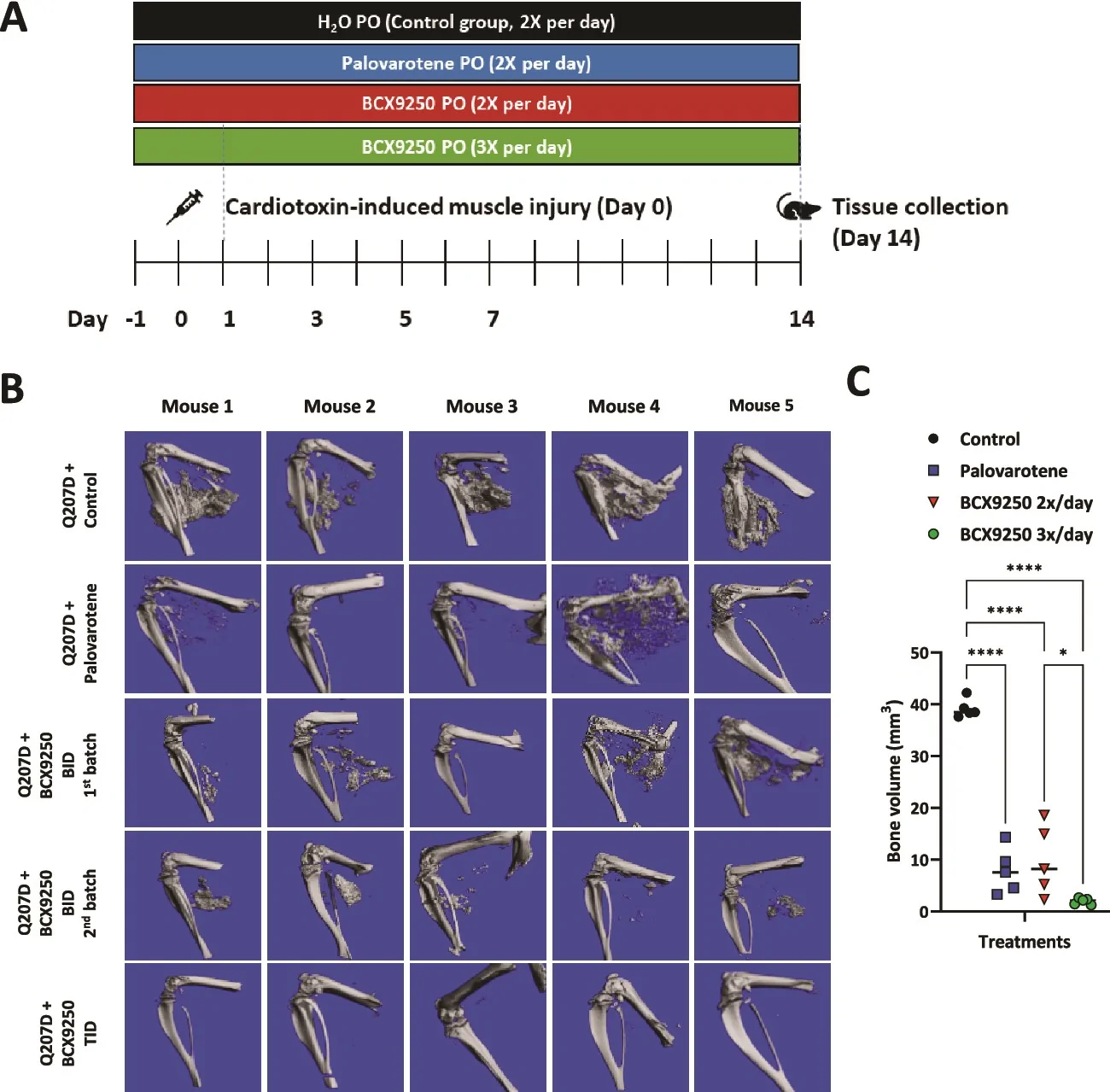
High-resolution imaging of heterotopic ossification (HO) in *Acvr1^Q207D^* mice by μCT. (A) Dosing schedule; (B) treatment of the *Acvr1^Q207D^* mice with palovarotene (2 times per day) or BCX9250 (2 or 3 times per day) for 14 d after cardiotoxin muscle injury; (C) quantification of HO volume in response to treatment with palovarotene or BCX9250. *N* = 5 mice for the control, palovarotene, and BCX9250 3×/day groups; *N* = 10 mice for the BCX9250 2×/day group; ^*^p = .0369; ^****^p < .0001.

**Figure 4:**
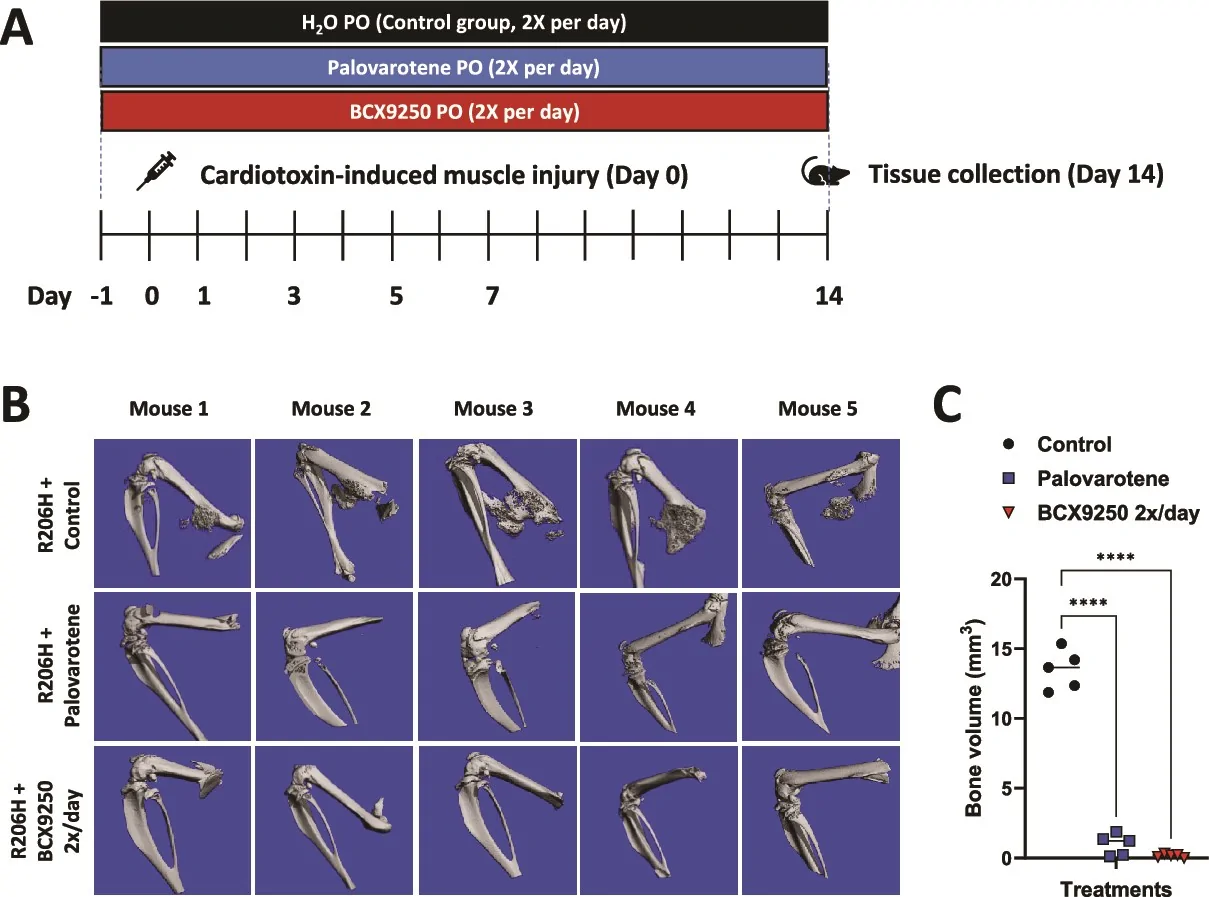
High-resolution imaging of heterotopic ossification (HO) in *Acvr1^R206H^* mice by μCT. (A) Dosing schedule; (B) treatment of the fibrodysplasia ossificans progressiva *Acvr1^R206H^* mouse model with palovarotene or BCX9250 (2 times per day) for 14 d after cardiotoxin muscle injury; (C) quantification of HO in response to treatment with palovarotene or BCX9250 2 times per day after cardiotoxin muscle injury in *Acvr1^R206H^* mouse. *N* = 5 mice per group; ^****^p < .0001.

HO was induced in mouse models by injecting cardiotoxin into the hind limb musculature and quantified using volumetric analysis of μCT-generated tomographic images. In the *Acvr1^Q207D^* mice, the HO observed in the control mice (38 mm^3^, Figure 3) was greater than that observed with *Acvr1^R206H^* mice (13 mm^3^; Figure 4), consistent with the strong activation of signaling expected for this model. Treatment with BCX9250 (BID for 15 d, N = 10 mice [first and second batch]; Figure 3A) resulted in significant abrogation of HO (9 mm^3^, *p* < .0001; Figure 3B and C), decreasing the HO to 24% of that observed in the control group. This reduction was similar in scale to that observed with palovarotene (8 mm^3^, *p* < .0001). Furthermore, increased administration of BCX9250 (PO administration, TID for 15 d) resulted in even greater inhibition of HO in *Acvr1^Q207D^* compared to BID administration (2 mm^3^, *p* = .0369; Figure 3B and C), reducing HO to 5% of that observed in the control group.

In the *Acvr1^R206H^* mice, the HO volume observed in control mice (13 mm^3^) was significantly reduced in mice treated with BCX9250 (PO administration, BID for 15 d; 1 mm^3^, *p* < .0001; Figure 4A). HO was reduced to 8% of that observed in the control group, similar in scale to the reductions observed with palovarotene treatment (2 mm^3^, *p* < .0001; Figure 4B). Thus, BCX9250 was an effective inhibitor of HO for both the ligand-dependent and the ligand-independent mouse models of aberrant pathway activation. Importantly, BCX9250 treatment did not elicit any observable side effects such as weight loss, alopecia, gross visual problems, or altered activity of the mice.

**Figure 5:**
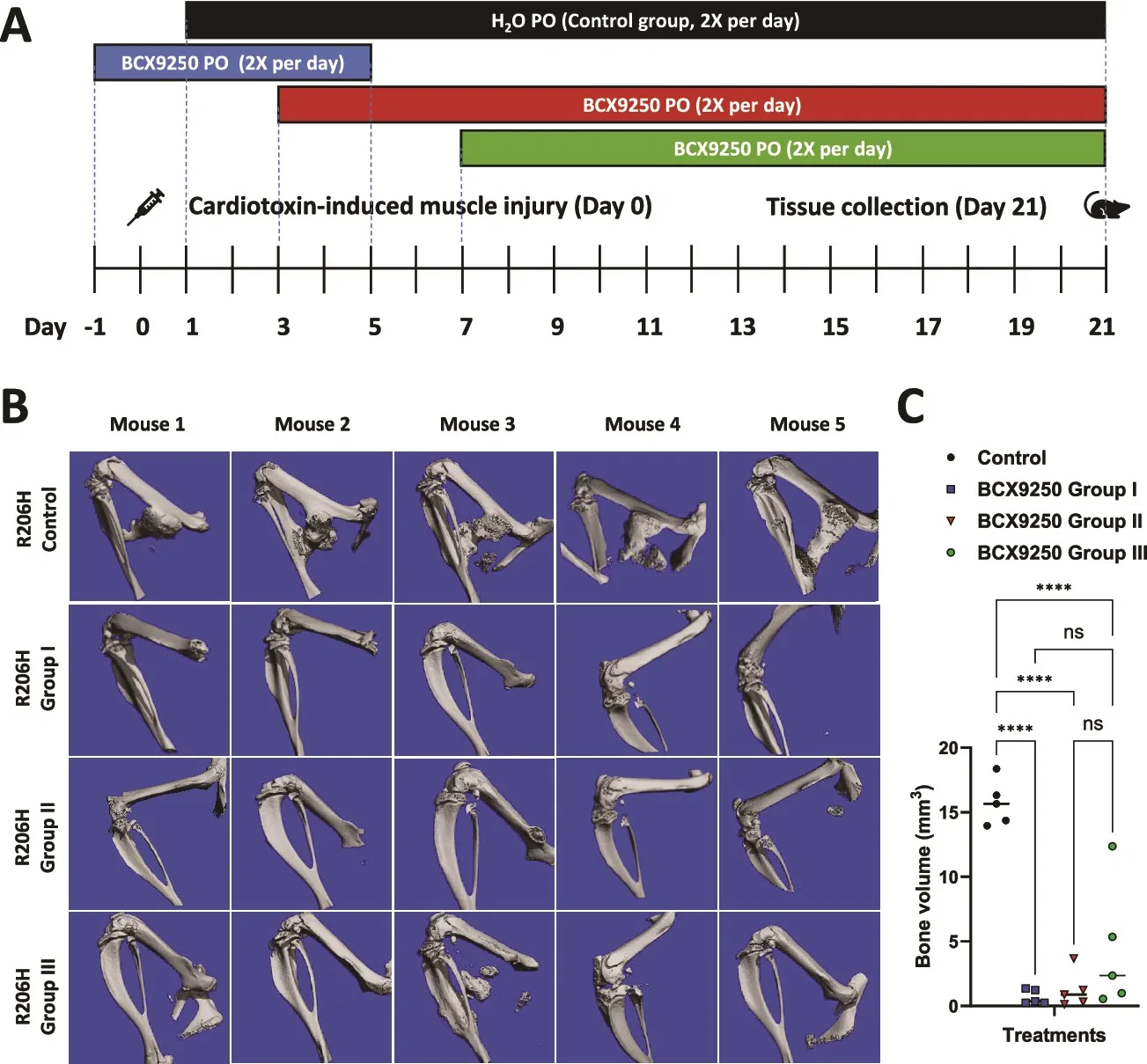
High-resolution imaging of heterotopic ossification (HO) in *Acvr1^R206H^* mice by μCT—early and delayed treatment regimens. (A) Dosing schedule; (B) treatment of the fibrodysplasia ossificans progressiva *Acvr1^R206H^* mouse model with BCX9250 (2 times per day for the indicated periods) and tissue collection 21 d after cardiotoxin muscle injury. BCX9250 (early treatment, group I): mice received cardiotoxin on day 0 and BCX9250 from day −1 through day 5; BCX9250 (delayed D3 treatment, group II): mice received cardiotoxin on day 0 and BCX9250 from day 3 through day 21; BCX9250 (delayed D7 treatment, group III): mice received cardiotoxin on day 0, BCX9250 from day 7 through day 21; (C) quantification of HO volume in response to early and delayed treatment with BCX9250 (2 times per day) after cardiotoxin muscle injury in *Acvr1^R206H^* mice. *N* = 5 mice per group; ^****^p < .0001.

Given the effectiveness of BID dosing of BCX9250 in the *Acvr1^R206H^* model, HO inhibition was assessed following administration under early- and delayed-treatment regimens (Figure 5A). Early oral BID dosing (starting on day −1, prior to cardiotoxin administration [group I], and continuing through day 5) significantly inhibited HO on day 21 (0.4 mm^3^) compared to the control group (16 mm^3^, *p* < .0001, Figure 5B and C). Delayed BID dosing (starting on day 3 [group II] or day 7 [group III] and continuing through day 21) also inhibited HO compared to the control group (group II, 0.3 mm^3^, *p* < .0001; and group III, 1.0 mm^3^, *p* < .0001). The efficacy of BCX9250 may have decreased slightly in group III, though these differences were not statistically significant compared to Groups I and II (*p* = .1872 and *p* = .3117, respectively).

### Treatment with BCX9250 reduces relapsed HO in the Acvr1 ^R206H^ FOP mouse model

**Figure 6:**
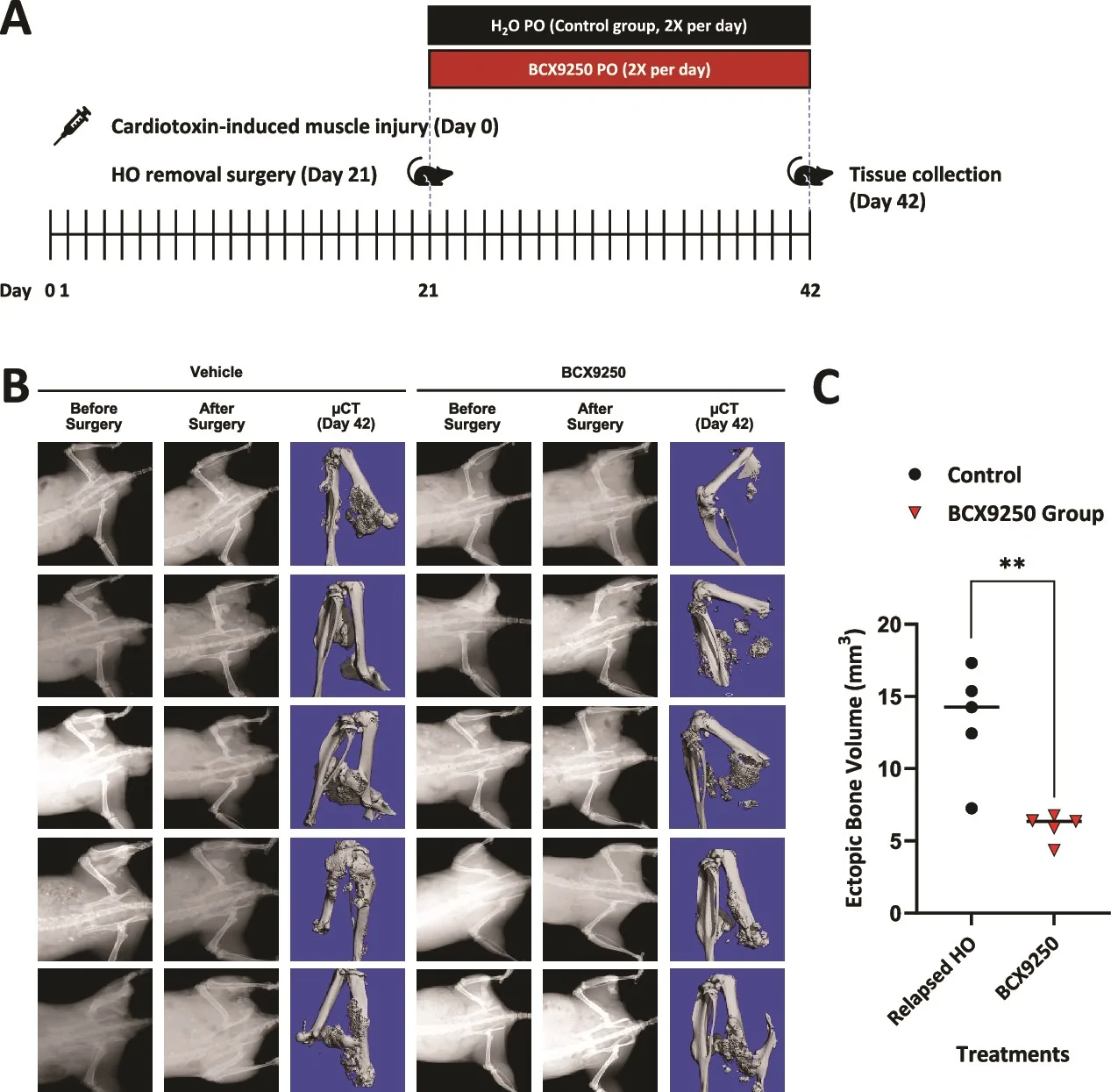
Treatment with BCX9250 reduces relapsed heterotopic ossification (HO) in an fibrodysplasia ossificans progressiva mouse model (Acvr1*^R206H^*). (A) Dosing schedule; (B) At day 21 after the cardiotoxin injection, the HO was surgically removed as confirmed using by X-ray imaging (comparison before and after surgery). Mice were treated with H_2_O per os (by mouth) (vehicle control group) or with BCX9250 (BCX9250 group) following surgery. After 21 d, mice were euthanized for μCT imaging; (C) HO volume was quantified. *N* = 5 mice per group; ^**^p < .01.

The significant reductions in HO observed with the delayed treatment regimen described above led us to evaluate BCX9250 as an inhibitor of relapsing HO after surgical removal of heterotopic bone in *Acvr1^R206H^* model mice (Figure 6A). For the relapsing HO mouse model, muscle injury was induced with cardiotoxin on day 0. On day 21 after induction, X-ray imaging was used to observe the heterotopic bone, which was then surgically excised. After closing the incision, X-ray imaging was used once again to confirm removal of the initial heterotopic bone so that new bone formation could be observed.

Oral BID administration of BCX9250 from day 21 (the day of surgery) through day 42 showed a significant inhibitory effect on HO (6.0 mm^3^) compared to the control group (13.3 mm^3^, *p* < .01, Figure 6B and C), yielding a 55% decrease in relapsing HO.

## Discussion

BCX9250 is a potent small-molecule inhibitor of ALK2. BCX9250 potently inhibited the activities of purified wild-type ALK2 and the FOP mutant ALK2^R206H^ in the binding assay, with IC_50s_ of 4.7 and 3.5 nM, respectively. BCX9250 also inhibits activin A-induced SMAD1 phosphorylation. Administration of BCX9250 in 2 mouse HO models results in a substantial reduction of trauma-induced HO. Furthermore, a short-term treatment in the early phase of trauma, or treatment delayed by up to 7 d with BCX9250, was equally effective in reducing HO in the Acvr^R206H^ mouse FOP model. In *Acvr1^Q207D^* and *Acvr1^R206H^* mice, BCX9250 inhibited HO to an extent equal to or greater than that provided by palovarotene, the only FDA-approved drug for the treatment of FOP. In a recurring HO model, after surgery and removal of the HO, BCX9250 protected the recurrent HO lesion. These data support the potential use of BCX9250 for the treatment of FOP.

The ca Acvr1^Q207D^ protein drives more extensive HO in response to injury. In the *Acvr1^Q207D^* mouse model, BID treatment with BCX9250 for 15 d was as effective as palovarotene at reducing HO. Furthermore, increasing the BCX9250 dosing frequency (TID) resulted in even greater suppression of HO. Efficacy was also observed in Acvr1^R206H^ mice expressing the ALK2^R206H^ protein, which produces lower levels of HO in response to injury. In this model, BID dosing with BCX9250 suppressed HO to the same extent as TID dosing in the *Acvr1^Q207D^* model, with reductions similar to those observed with palovarotene treatment. Given the effectiveness of BCX9250 in the *Acvr1^R206H^* mouse model, we evaluated BID dosing across treatment timing and duration. We found that oral administration of BCX9250 was effective in reducing HO across a wide range of treatment regimens, with dosing beginning as early as 1 d prior to cardiotoxin-induced muscle injury or as late as 7 d after injury. Treatment durations of 6 d, 14 d, and 18 d were similarly effective in this mouse model. The substantial reductions to HO observed with the brief preventive treatment are notable, given that the mice received no BCX9250 for most of the study.

The severity of the recurrence of HO observed in FOP patients precludes surgical interventions to remove primary lesions or address other medical issues.^10^^,^^11^^,^^39^^,^^40^ Thus, drug treatments that minimize recurrent HO would be of great clinical value, providing a treatment option that could readily improve a patient’s mobility and quality of life, while significantly expanding the potential applications of drug candidates.^41^ The success of our delayed treatment protocol in the *Acvr1^R206H^* mouse model of FOP motivated the evaluation of BCX9250 as a treatment for HO recurrence. In a mouse model of relapsing HO after surgery, BID dosing of BCX9250 following surgical excision of the primary lesion reduced HO recurrence by 60% compared with the control group, though more quantitative methods for comparing lesion volumes before and after surgery are needed to fully interpret these effects. To our knowledge, this is the first experimental approach to address recurrent HO after surgical removal.

The selectivity study showed that, in addition to ALK2, BCX9250 also potently inhibited several other wild-type protein kinases, including LCK, RIPK, and SRC. The present study cannot rule out the contribution of the other protein kinases in BCX9250’s anti-HO effects. BCX9250 also inhibited ALK1-driven transcriptional activity, though the inhibition was about 4-fold less potent than ALK2. ALK1 regulates endothelial cell proliferation and migration and is crucial for angiogenesis. The loss of ALK1-mediated signaling is likely to cause telangiectasias, introducing a risk of telangiectasia with BCX9250 treatment and ALK1 inhibition.

Deletion of ALK2 in mice leads to downregulation of hepcidin expression and moderate iron overload, while the overload is more severe upon deletion of ALK3.^36^ However, 14-d treatment with BCX9250 in Acvr1^Q207D^ mice resulted in about a 1-fold increase in plasma hepcidin levels. No inflammatory signs were observed in BCX9250 treated mice. Use of the Acvr1^Q207D^ mouse model may complicate this picture by reducing the baseline iron levels prior to inhibition, making the system more sensitive to other effects. The unexpected increase in hepcidin and the potential for BCX9250 to cause anemia could be a concern for long-term use of the drug in the treatment of FOP. Thus, clinical development of BCX9250 should include careful assessment of iron homeostasis following BCX9250 administration.

Notably, published preclinical studies of BLU-782 (IPN60130) identified a short treatment window following injury, with prevention of HO observed when treatment was delayed for 2 d, whereas delays of 4 d or longer abrogated HO prevention.^36^^,^^42^ In the present study, BCX9250 significantly reduced HO in Acvr1R206H mice when treatment was initiated 3 or 7 d after cardiotoxin-induced muscle injury. Although differences in experimental models, dosing regimens, and study design preclude direct comparison between these compounds, the efficacy observed with delayed BCX9250 treatment supports further investigation of its activity during different stages of HO development.

Our results with BCX9250 showed significant reductions in HO. Compared to palovarotene, BCX9250 directly targets ALK2. Because palovarotene acts indirectly (via the retinoid pathway) rather than directly on the core mutant signaling node (ALK2), it may have limited efficacy or require combinatorial approaches. Indeed, targeting ALK2 could offer greater specificity and perhaps fewer off-target effects than a broad-spectrum modulator like a retinoid. We showed that mice dosed with BCX9250 had no weight loss and no decline in normal activities, which is encouraging. While our data clearly demonstrate the efficacy of BCX9250 as a treatment in these mouse models—effectively suppressing primary and recurrent HO—further studies are required to assess the long-term effects of BCX9250 dosing.

## Data Availability

All data are incorporated into the article and its online supple-mentary material.
